# Supplementary material for: Examining women’s choice between home and institutional births: Insights from the Salud Mesoamérica Initiative (SMI)
Source: PLoS One. 2026 Mar 25;21(3):e0345813. doi: 10.1371/journal.pone.0345813 (PMC13016324; doi:10.1371/journal.pone.0345813)
Supplement: S1 Table — List of the corresponding household survey questions related to the four domains in institutional birth. (DOCX) [file pone.0345813.s001.docx]

**S1 Table. Variables related to quality of care - analysing institutional birth.**

| **Domain** | **Label** |
| --- | --- |
| 1. Respectful treatment | Did health personnel allow you to select the sex of the person who attended the birth? |
|  | Did the health staff allow you to wear the clothes you wanted? |
|  | Did the health personnel explain to you what they were doing? |
|  | Did the health personnel treat you with respect? |
|  | Did the health personnel deliver the placenta to you? |
| 2. Effective communication | Did health personnel speak to you in your own language? |
|  | Did you understand what the health personnel explained to you? |
| 3. Accompaniment | Did health personnel allow you to be accompanied by a family member or a midwife? |
| 4. Recommended practices | Did the health personnel allow you to drink the liquids and take the remedies you wanted? |
|  | Did the health staff ensure that you were sufficiently warm? |
|  | Was the place clean? |
|  | Did health personnel allow you to choose the position for delivery? |
