# Supplementary material for: Examining women’s choice between home and institutional births: Insights from the Salud Mesoamérica Initiative (SMI)
Source: PLoS One. 2026 Mar 25;21(3):e0345813. doi: 10.1371/journal.pone.0345813 (PMC13016324; doi:10.1371/journal.pone.0345813)
Supplement: S2 Table — List of the corresponding household survey questions related to the six domains in home birth. (DOCX) [file pone.0345813.s002.docx]

**S2 Table. Variables related to reasons for home birth- analysing home birth.**

| **Domain** | **Label** |
| --- | --- |
| 1. Knowledge | 1. No one advised me to go to a health center for childbirth |
| 2. Accessibility | 2. The center is too far away |
| 2. Accessibility | 3. I could not afford transportation |
| 2. Accessibility | 4. I could not find transportation |
| 2. Accessibility | 5. I did not have time to travel |
| 1. Knowledge | 6. I did not know where to go |
| 3. Infrastructure | 7. The center's facilities are inadequate |
| 3. Infrastructure | 8. The center does not have enough medications |
| 3. Infrastructure | 9. The center is not well-equipped |
| 4. Respectful treatment and Effective communication | 10. It is difficult to deal with the staff at the center |
| 4. Respectful treatment and Effective communication | 11. The center staff is not well-informed |
| 4. Respectful treatment and Effective communication | 12. I do not trust the staff |
| 4. Respectful treatment and Effective communication | 13. I was treated badly at the center in the past |
| 5. Family | 14. My partner or husband did not allow me to go |
| 5. Family | 15. Another family member did not allow me to go |
| 4. Respectful treatment and Effective communication | 16. I tried to go to a center, but I was denied care |
| 6. Cultural preferences | 17. I did not want to go alone |
| 6. Cultural preferences | 18. I wanted a traditional midwife to accompany me |
| 6. Cultural preferences | 19. I prefer to give birth with a traditional midwife |
| 6. Cultural preferences | 20. Religious/cultural beliefs |
| 3. Infrastructure | 21. The health service is too far, and I had nowhere to stay (there was no maternal home nearby) |
| 2. Accessibility | 22. The health service charges for childbirth. How much? Specify the amount: |
| 6. Cultural preferences | 23. I preferred to give birth at home or in another house |
| 3. Infrastructure | 24. There was no staff at the center when I visited |
|  | 25. Other: Specify  26. Does not know  27. Does not answer |
